# Reserving the right to change the intestinal stem cell model

**DOI:** 10.1016/j.stem.2020.02.003

**Published:** 2020-03-05

**Authors:** Simon J. Leedham

**Affiliations:** Intestinal Stem Cell Biology Laboratory, Oxford Centre for Cancer Gene Research, Wellcome Trust Centre for Human Genetics, University of Oxford, Roosevelt Drive, Oxford, OX3 7BN, United Kingdom

## Abstract

The debate concerning the existence of ‘active’ and ‘reserve’ stem cell populations in the intestinal epithelium has been ongoing since 1977. A new paper brings some closure to this argument, by showing that all intestinal regeneration arises from daughter cell dedifferentiation. This marks the coming-of-age of the regenerative stem cell plasticity model.

Some scientific observations are ahead of their time. A putative intestinal crypt-based columnar stem cell population was proposed by Cheng and Leblond in 1974 (Cheng and Leblond, 1974). It was a further 33 years before Barker et al demonstrated multipotency and self-renewal in this population and identified *Lgr5* as a cell marker (Barker et al., 2007). Similarly, the concept of a label retaining, reserve stem cell population, located at the +4 position in the intestinal crypt, was proposed by Chris Potten as long ago as 1977 (Potten, 1977). A debate about the existence and role of these cells has continued ever since. Tian et al contributed to this discussion in 2011, using ingenious new mouse tools to demonstrate the efficient replacement of damaged *Lgr5+* CBC’s from cells above the crypt base (Tian et al., 2011). However, the scope of the debate shifted in 2013, when the work of Buczacki *et al.* altered the paradigm (Buczacki et al., 2013). They showed that the fabled +4 cell population were in fact post-mitotic progenitors - daughter cells arising from CBC stem cell division and progressing towards a secretory cell fate, rather than a defined, self-renewing stem cell pool that could be tagged by specific markers. Since then, a host of papers have demonstrated cell plasticity and dedifferentiation capacity across a range of cell types in response to crypt damage or stem cell depletion (de Sousa and de Sauvage, 2019). In this edition of *Cell Stem Cell,* the Shivadasani and Clever’s groups seek to definitively address the active and reserve stem cell population hypothesis, by showing that all crypt regeneration arises through dedifferentiation of stem cell progeny (Murata et al, 2020). This paper marks the coming-of-age of the regenerative stem cell plasticity model, and brings some final closure to a 40-year old debate.

The work was principally undertaken in mouse models, with intestinal epithelial integrity challenged either by γ-irradiation or diptheria toxin-induced depletion of *Lgr5+* stem cells, two established injurious models that trigger a predominantly epithelial compartment adaptive reprogramming response. Fluorescent lineage markers were used to meticulously quantify the contribution of cell dedifferentiation to regeneration of the *Lgr5+* cell population. This was assessed in steady state conditions and following combination with models to manipulate the Notch signalling pathway *(Atoh* and *Rbpj* knockout mice, which induce secretory and enterocyte cell depletion respectively). In all cases, regenerated stem cells arose predominantly from ISC daughter cells, with robust contribution from both secretory and enterocyte cell populations when the alternative population was depleted. Previous work had implicated the ISC-restricted transcription factor and wnt target, *Ascl2,* as a key regulator of stem cell survival (van der Flier et al., 2009) and compound heterozygote models were used to generate fluorescent marker and epitope tagged *Ascl2* knockout mice. Interestingly, these animals had no immediate homeostatic phenotype, but quantitative modelling of labelled cells over time showed that *Ascl2* loss bestowed a small but impactful negative selective advantage, skewing neutral drift and favouring mutant stem cell replacement. It was only after injury that the regenerative indispensibility of *Ascl2* became apparent with knockout animals succumbing soon after irradiation or stem cell depletion. Next, the team used a clever labelling strategy to mark *Ascl2* cells before depleting the *Lgr5+* cell population. Rapid re-activation of *Ascl2* expression was seen in a host of cells in the upper crypt (above cell +5), as the loss of the stem cell compartment triggered a progenitor cell response, and these cells, ‘caught-in-the act’ of de-differentiation, were collected to undergo single cell transcriptome interrogation. Perhaps unsurprisingly, a number of cells revealed a broad range of concomitant gene expression, from established stem cell genes through to enterocyte and goblet cell markers, consistent with the transition of cells from mature to de-differentiated state. However, genes previously shown to be upregulated in the response to mucosal barrier breach and established as fetal epithelial cell markers (Yui et al., 2018) and/or associated with YAP/TAZ signalling (Gregorieff et al., 2015), did not concentrate in these transitional cells. Finally, the paper assessed the cell surface receptor targets of *Ascl2* transcription and identified potential functional significance in the upregulation of interleukin receptor 11 (Il11Ra1) in de-differentiating cells, intimating that enhanced cytokine signalling sensitivity may contribute to the regulating the response of these reprogrammed cells in the regenerating crypt.

This is an important paper, both for the questions it answers, and the questions it raises. That is has taken 40 years, the development of a suite of ingenious transgenic animals, organoid culture systems and state-of-the art genomics to finally refine the ‘reserve’ stem cell model is perhaps a testament both to the prescience of the original observation and the perseverance, ingenuity and scientific curiousity of current researchers in the field. This demonstration of *Ascl2* as an epithelial cell-intrinsic regulator of cell plasticity marks the transition of the query in the intestinal epithelial stem cell plasticity field from ‘whether and which?’ to ‘how and when?’. There is much to learn. What mechanism senses stem cell loss in the crypt? What cell-signalling changes trigger and drive such a rapid and profound adaptive reprogramming response in post-mitotic cells? What is the contribution of the different cell compartments in the dynamic response to injury? These questions may impact upon the observation of the lack of contribution of developmental gene signatures and YAP/TAZ signalling to the cells transitioning from differentiated to stem state. This contrasts with recent findings, including those of Yui *et al,* who showed that epithelial reprogramming occurred via a fetal epithelial state, regulated by ECM remodelling and mechanotransduction (Yui et al., 2018). This apparent incongruity may be explained by the nature of the injuries applied in these different papers and the temporality of the observations made. Here, the injury is directed at the epithelial cell compartment, predominantly through depletion of the *Lgr5* stem cell population, and the changes invoked in epithelial cells occur within 48 hours. This targeted approach is mechanistically ideal for studying the epithelial compartment response to stem cell loss, but does not necessarily reflect a defined human disease correlate. Yui *et al.* used Dextran sodium sulphate colitis to induce barrier breach, with concomitant innate immune cell infiltration, stromal cell activation and resultant extracellular matrix remodelling. Changes took up to a week to establish. It is plausible that ECM remodelling and mechanotransduction, evoked by this multi-compartmental regenerative response, is required for fetal reprogramming, and that this is less likely to contribute to the response seen following targeted and specific epithelial stem cell depletion. Defining the spatio-temporal changes and the contribution of different cell compartments in sensing and responding to damage will be a research priority for the field in future.

The intestinal mucosa has evolved to generate an effective and rapid response to injury and barrier breach, and this response is underpinned by a spectrum of epithelial cell plasticity, driven by cell-endogenous and exogenous microenvironmental cues that we are yet to fully comprehend. Understanding this is key to potential future therapeutic manipulation - either temporarily enhancing the dedifferentiation response to promote intestinal regeneration in inflammatory bowel disease or trying to suppress cell plasticity in cancer stem cells.
